# Amino Acids Regulate Cisplatin Insensitivity in Neuroblastoma

**DOI:** 10.3390/cancers12092576

**Published:** 2020-09-10

**Authors:** Venugopal Gunda, Anup S. Pathania, Srinivas Chava, Philip Prathipati, Nagendra K. Chaturvedi, Don W. Coulter, Manoj K. Pandey, Donald L. Durden, Kishore B. Challagundla

**Affiliations:** 1 Department of Biochemistry and Molecular Biology & The Fred and Pamela Buffett Cancer Center, University of Nebraska Medical Center, Omaha, NE 68198, USA; venu.gunda@unmc.edu (V.G.); anup.pathania@unmc.edu (A.S.P.); srinivas.chava@unmc.edu (S.C.); 2 Laboratory of Bioinformatics, National Institutes of Biomedical Innovation, Health and Nutrition, 7-6-8 Saito-Asagi, Ibaraki City, Osaka 567-0085, Japan; philip.prathipati@gmail.com; 3 Department of Pediatrics, Division of Hematology/Oncology, University of Nebraska Medical Center, Omaha, NE 68198, USA; nchaturvedi@unmc.edu (N.K.C.); dwcoulter@unmc.edu (D.W.C.); 4 Department of Biomedical Sciences, Cooper Medical School of Rowan University, 401 South Broadway, Camden, NJ 08103, USA; pandey@rowan.edu; 5 Division of Pediatric Hematology and Oncology, Department of Pediatrics, Moores Cancer Center, University of California, San Diego, 3855 Health Science Drive, MC-0815, La Jolla, CA 92093, USA; ddurden@health.ucsd.edu; 6 SignalRx Pharmaceuticals, Inc. 8330, Loveland Drive, Omaha, NE 68124, USA; 7 The Children’s Health Research Institute, University of Nebraska Medical Center, Omaha, NE 68198, USA

**Keywords:** neuroblastoma, MYCN, cisplatin-sensitivity, metabolism, amino acids, hydroxychloroquine

## Abstract

**Simple Summary:**

Neuroblastomas mostly show poor response to the Cisplatin therapy. Amino acids serve as building blocks for proteins, which are acquired either through diet or protein breakdown. Our study reveals high amino acid pools and dependence of Cisplatin-tolerant neuroblastomas cells on amino acids for their survival, especially, in drug treated conditions. Our study also demonstrates that response of neuroblastomas to Cisplatin can be improved by decreasing cellular amino acid levels either by reducing amino acid supplements or by applying autophagy inhibitor, Hydroxychloroquine. Thus, our findings establish that neuroblastomas can be sensitized to Cisplatin by targeting amino acid metabolism.

**Abstract:**

Neuroblastoma are pediatric, extracranial malignancies showing alarming survival prognosis outcomes due to their resilience to current aggressive treatment regimens, including chemotherapies with cisplatin (CDDP) provided in the first line of therapy regimens. Metabolic deregulation supports tumor cell survival in drug-treated conditions. However, metabolic pathways underlying cisplatin-resistance are least studied in neuroblastoma. Our metabolomics analysis revealed that cisplatin-insensitive cells alter their metabolism; especially, the metabolism of amino acids was upregulated in cisplatin-insensitive cells compared to the cisplatin-sensitive neuroblastoma cell line. A significant increase in amino acid levels in cisplatin-insensitive cells led us to hypothesize that the mechanisms upregulating intracellular amino acid pools facilitate insensitivity in neuroblastoma. We hereby report that amino acid depletion reduces cell survival and cisplatin-insensitivity in neuroblastoma cells. Since cells regulate their amino acids levels through processes, such as autophagy, we evaluated the effects of hydroxychloroquine (HCQ), a terminal autophagy inhibitor, on the survival and amino acid metabolism of cisplatin-insensitive neuroblastoma cells. Our results demonstrate that combining HCQ with CDDP abrogated the amino acid metabolism in cisplatin-insensitive cells and sensitized neuroblastoma cells to sub-lethal doses of cisplatin. Our results suggest that targeting of amino acid replenishing mechanisms could be considered as a potential approach in developing combination therapies for treating neuroblastomas.

## 1. Introduction

Neuroblastomas are extracranial tumors arising in adrenal glands, most commonly found in infants and young children. Rarely, children older than ten years are diagnosed with these extracranial neuroblastoma tumors that originate from neuroblasts [1,2]. Overall, five-year survival outcomes have been alarming, with around 40% or less survival observed in children diagnosed with neuroblastoma. This is an indicative of high mortality, especially in neuroblastoma patients diagnosed with high-risk tumors who are predicted to have poor survival outcomes due to the aggressive growth and therapy resilience prevalent in high-risk patient tumors [3,4,5]. Standard therapies prescribed for neuroblastoma patients include a combination of chemotherapy, radiation therapy and immunotherapies, along with stem cell therapies and surgery being mostly opted for high-risk neuroblastoma patients [6,7]. Despite the strategic application of clinically available chemotherapy agents in combination therapeutic approaches, neuroblastoma patients often show disease recurrence due to therapy resistance which is commonly evident in the poor responders [8,9]. Thus, a deep understanding of the resistance mechanisms which counteract the therapeutic outcomes would enable in developing alternative strategies to improve the therapeutic outcomes in neuroblastoma patients [10].

Cisplatin (CDDP) is applied as a frontline chemotherapy agent for neuroblastoma patients, including the high-risk cases [11]. Like other platinum-based chemotherapeutic agents, CDPP’s efficacy relies on DNA damaging effects, which often fails due to the innate and acquired resistance prevalent among tumor cells [12]. Cell line models of neuroblastoma exhibit heterogeneity in their CDDP response due to the resistance mechanisms underlying their drug sensitivity. Availability of the sensitive and resistant, neuroblastoma cell lines models for CDDP response have enabled in deciphering the role of CDDP-resistance mechanisms in neuroblastoma. Elaboration of CDDP resistance mechanisms using tumor cell lines has revealed that survival of tumor cells in cisplatin-treated conditions primarily relies on adaptive mechanisms that enable tumor cells in switching to oncogenic mode which enable evasion of cytotoxicity in drug treated conditions [13]. Thus, utilizing cell lines as models mechanisms mediated through intra-tumoral oncogenes, mutations and signaling cascades, as well as extra-tumoral factors, such as stromal components and non-cellular components, have been identified to affect CDDP sensitivity [10,14,15,16,17]. Multiple mechanisms mediated by oncogenes, such as exosomal-microRNAs and cell signaling pathways, were identified to promote CDDP-insensitivity in neuroblastoma [10,18,19,20]. The oncogenes, including v-myc avian myelocytomatosis viral oncogene neuroblastoma derived homolog (MYCN) and Hypoxia-inducible factor (HIF)-1∝, were shown to regulate metabolism and mediate CDDP-resistance in neuroblastoma [21,22,23].

Metabolic rewiring is an effective phenomenon opted by tumor cells in evading therapy response. Recent studies have demonstrated that oncogene-mediated metabolism augments tumor growth in neuroblastoma [24]. Direct metabolic dependencies of tumor cells are being explored to exploit these dependencies as novel therapeutic targets for sensitizing neuroblastoma to therapeutic agents [25]. In similar lines, the pathological role of metabolic pathways, including purine metabolism, sphingolipid metabolism, and glutathione metabolism, has been enumerated in regulating neuroblastomas’ response to chemotherapeutic agents, such as Thioguanines [26], fenretinide [27], and Etoposide [28], respectively. Metabolic pathways involving amino acids also play key role in promoting cancer [29]. Metabolism of Serine, Glycine, Glutamine, and Tryptophan metabolites have been shown to promote cellular growth in neuroblastoma models [24,30,31]. Tumor cells maintain their amino pools through uptake of exogenous amino acids which is under tight regulation through cellular receptor expression and mechanistic Target of Rapamycin kinase (mTOR) [32]. In addition to the uptake of amino acids, endogenous protein degradation through autophagy, especially in stress conditions, such as drug treatments, enables cancer cells in maintaining their amino acid pools [33]. Identifying such precise, metabolic readouts in drug-resistant tumor cells facilitates targeting of metabolic pathways, especially in drug-induced scenarios, such as CDDP-induced resistance. In the current study, we applied a metabolomics-based approach to decipher the role of amino acid metabolism in regulating cisplatin-insensitivity of neuroblastoma using cell line models. Our study establishes the reliance of CDDP-insensitive cells on amino acids for surviving in cisplatin-treated conditions.

## 2. Results

### 2.1. Cisplatin-Insensitive Neuroblastoma Cells Exhibit Metabolic Plasticity

**Figure 1:**
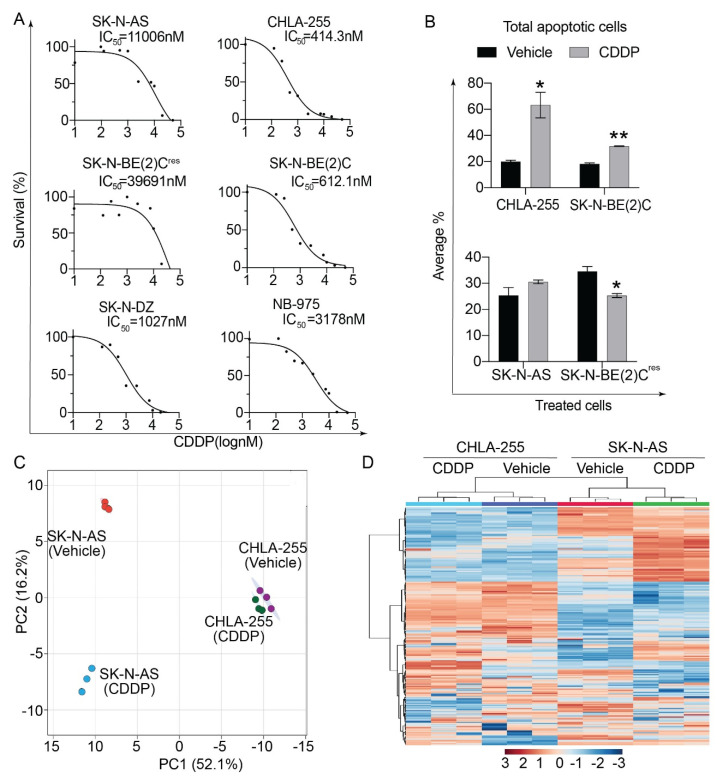
Cisplatin (CDDP) treatment alters metabolism in neuroblastoma. (**A**) The half-maximal inhibitory concentration (IC_50_) curves showing different survival response in neuroblastoma cells upon cisplatin (CDDP) treatment. Each data point represents mean ± SE values from three biological replicates. (**B**) Bar graphs showing percentage of total (late and early) apoptotic cells after 24 h treatments with either vehicle or CDDP treatments in neuroblastoma cells. * and ** indicate *p* < 0.05 and *p* < 0.01 obtained from three biological replicate data derived using Student’s *t*-test. (**C**) Principal Component Analysis (PCA) plot showing the segregation of vehicle and cisplatin (CDDP) treated cells based on their metabolite profiles. Each colored circle represents a biological replicate of the treatment condition. (**D**) Heatmap showing the clustering of vehicle and cisplatin (CDDP) treated groups. Colored cells in each row represent metabolites, and each column represents a sample. The scale for the heatmap is shown under the heatmap.

In order to sort out the CDDP-sensitive and -insensitive neuroblastoma cell line models, we evaluated the survival response of different neuroblastoma cells, including SK-N-BE(2)C, SK-N-DZ, CHLA-255, SK-N-AS, and SK-N-BE(2)C^res^—a CDDP-resistant model developed in our laboratory—, and murine NB-975 cells, upon treatment with various concentrations of CDDP. As shown in Figure 1A, SK-N-BE(2)C^res^, SK-N-AS, and murine NB-975 cells showed less sensitivity to CDDP, whereas CHLA-255, SK-N-BE(2)C, and SK-N-DZ showed higher sensitivity to CDDP as evident from the differences in cell survival upon drug treatments. We confirmed the difference in sensitivities of our model cell lines by analyzing percentage of the late and early apoptotic cells detected after CDDP-treatments using Annexin-V staining and flow cytometric analyses. As shown in Figure 1B, cisplatin-sensitive CHLA-255 and SK-N-BE(2)C cells showed significantly high percentage of apoptotic cells in CDDP-treatments, in comparison to the vehicle treated conditions. In contrast to sensitive cells, apoptotic phenotype was not altered in CDDP-treated SK-N-AS cells in comparison to the vehicle treatments. Intriguingly, the percentage of apoptotic cells was low in CDDP-treated SK-N-BE(2)C^res^ cells in comparison to their vehicle treated counterparts (Figure 1B). Hereafter we referred SK-N-AS cells as CDDP-insensitive and CHLA-255 as CDDP-sensitive cells throughout the rest of the manuscript. To characterize the metabolic changes upon CDDP-treatment, we used SK-N-AS (CDDP-insensitive) and CHLA-255 (CDDP-sensitive) cell lines as innately insensitive and sensitive CDDP-cell lines models for polar metabolomic analysis. We present the metabolite differences between control and CDDP treated cells in the form of the Principal Component Analysis (PCA) plot. As evident from the PCA plot in Figure 1C, CDDP-sensitive CHLA-255 cells and CDDP-insensitive, SK-N-AS cells separated on the PC1 axis, indicating metabolic differences among these cells. Furthermore, the segregation of the vehicle and CDDP-treated, SK-N-AS and CHLA-255 cells was different along the PC2 axis. The vehicle and CDDP-treated, cisplatin-sensitive CHLA-255 cells showed relatively minor change in their polar metabolic profile, indicated by the close clustering of the vehicle and CDDP-treated groups on the PCA plot (Figure 1C). In contrast to the sensitive cells, vehicle and CDDP-treated cisplatin insensitive, SK-N-AS cells showed higher separation along the PC2 axis (Figure 1C), indicating significant contrast in the metabolite pools of SK-N-AS cells upon CDDP-treatment. In addition to the PCA analysis, the metabolite changes presented in the form of heatmap analysis also indicated that sensitive and insensitive neuroblastoma cells exhibited a contrasting trend in metabolic profiles upon CDDP-treatment (Figure 1D). Based on metabolic profile in the heatmap, vehicle-treated, sensitive cells and insensitive cells clustered closely with their respective CDDP-treated groups. Further, heatmap analysis revealed that the relative alterations in the individual metabolite levels were more drastic in SK-N-AS cells upon treatment with CDDP.

### 2.2. Cisplatin Treatment Alters Amino Acid Metabolism in Drug Insensitive Neuroblastoma Cells

**Figure 2:**
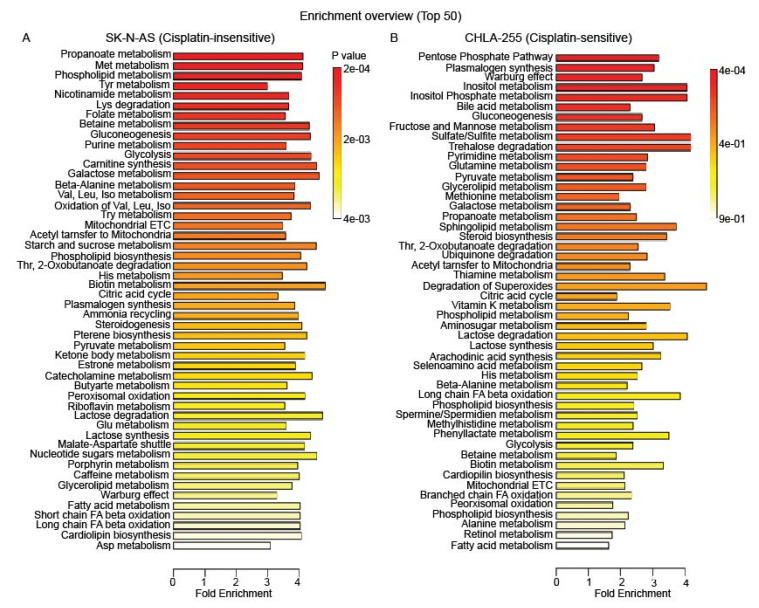
Cisplatin treatment affects metabolic pathways in neuroblastoma cells. The pathway impact analyses showing top 50 metabolic pathways altered in (**A**) cisplatin-insensitive, SK-N-AS and (**B**) cisplatin-sensitive, CHLA-255 cells upon cisplatin treatment. Three biological replicates were used in each condition. The scale under the plot, indicates fold enrichment for each plot, whereas the color scale adjacent to the respective plot indicates *p*-values for the impacted pathways.

We validated the key differences in metabolic pathways altered upon drug treatment in CDDP-insensitive and sensitive neuroblastoma cells. Pathway enrichment analysis using MetaboAnalyst revealed the top 50 metabolic pathways altered in CDDP-treated insensitive, SK-N-AS and sensitive, CHLA-255 cells compared to their respective vehicle-treatments (Figure 2). Metabolic pathways encompassing amino acids, vitamins, carbohydrates (like Glycolysis, Warburg effect), sugar and nucleotide metabolism, mitochondrial metabolism (including branched amino acid metabolism, Citric acid cycle, and β-oxidation), and peroxisomal metabolism were significantly altered in SK-N-AS cells upon treatment with CDDP. Metabolic pathways in particular central carbon and nucleotide related (Pentose phosphate pathway, Warburg effect, gluconeogenesis, fructose and mannose, pyrimidine and pyruvate metabolic pathways), and lipid metabolic pathways (inositol, bile salts, glycolipid, sphingolipid, and phospholipid metabolic pathways) were among the most significantly altered pathways in CHLA-255 cells upon CDDP treatment. Thus, alterations in metabolic pathways observed in CDDP-treated, sensitive, CHLA-255 cells were distinct from CDDP-treated, insensitive, SK-N-AS cells based on the differences in the most significantly affected pathways. Among the top 50 hits, there was a high frequency of alterations in amino-acid metabolic pathways in CDDP-insensitive, SK-N-AS cells. Therefore, we conclude that the metabolism of Methionine, Tyrosine, Lysine, Valine, Leucine, Isoleucine, Tryptophan, Threonine, Histidine, and Glutamate (sorted in descending order) were significantly altered in insensitive, SK-N-AS cells upon CDDP-treatment, which were not noticed in sensitive, CHLA-255 cells (Figure 2).

### 2.3. Cisplatin-Insensitive Neuroblastoma Cells Upregulate Amino Acid Metabolism

**Figure 3:**
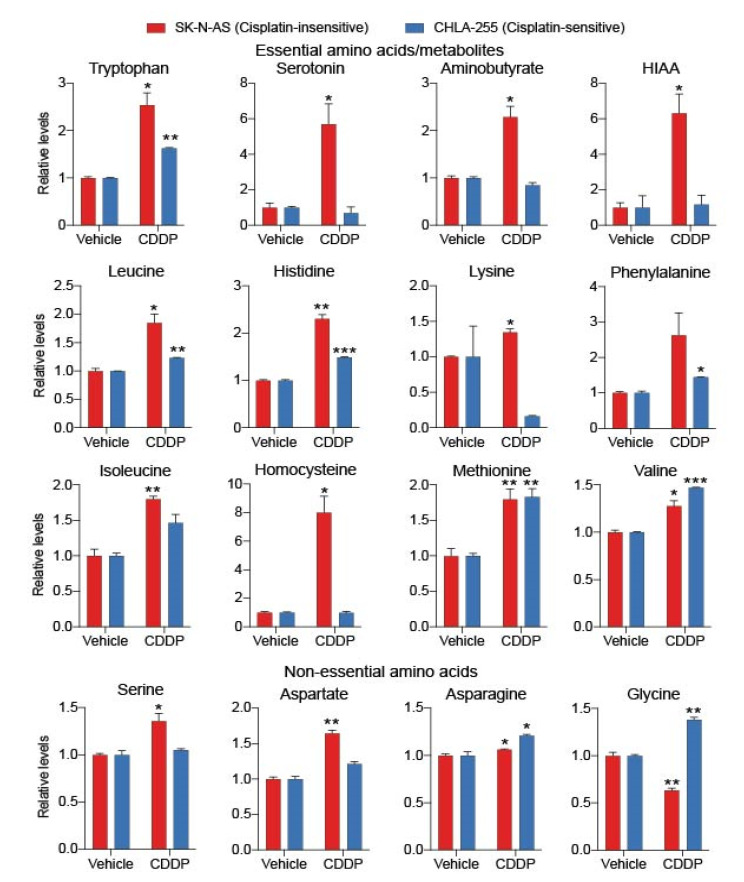
Amino acid metabolism was upregulated in cisplatin-insensitive neuroblastoma cells. Bar graphs showing relative quantification of amino acid levels in vehicle and cisplatin-treated insensitive SK-N-AS and sensitive CHLA-255 cells. Each bar represents mean ± SE of data obtained from three biological replicates. *, ** and *** indicate *p* < 0.05, *p* < 0.01 and *p* < 0.001, respectively, which were obtained through Student’s *t*-test by comparing the relative metabolite of the treated cell line with its respective vehicle, control value.

We quantified individual amino acid metabolite levels in neuroblastoma cells upon treatment with cisplatin as presented in Figure 3. Our comparative analysis revealed an upregulation of eight essential amino acids (Histidine, Isoleucine, Leucine, Lysine, Methionine, Phenylalanine, Tryptophan, and Valine) in CDDP-insensitive, SK-N-AS cells upon treatment with CDDP (Figure 3). The upregulation of essential amino acids, and their derived metabolites was noticed in only few metabolites in CDDP treated, sensitive CHLA-255 cells. Thus, the frequency of upregulation in amino acids and their metabolites was relatively low in sensitive cells compared to SK-N-AS cells (Figure 3). We also observed that Tryptophan levels were significantly higher in both sensitive and insensitive neuroblastoma cells. However, Tryptophan derived metabolites Serotonin, Aminobutyrate, and 5-Hydroxyindole acetic acid (HIAA) were significantly upregulated only in CDDP-treated, insensitive but not in CHLA-255 cells. Similarly, the relative increase of Leucine, Histidine, Lysine, Isoleucine, Homocysteine, Serine, and Aspartate levels was also higher in CDDP-treated, insensitive, SK-N-AS cells compared to CDDP-treated, sensitive cells. In conclusion, out of 16 amino acids that were altered, we observed only three amino acids (Glycine, Asparagine, and Valine) to be relatively enhanced in CDDP treated sensitive cells compared to CDDP-treated insensitive cells (Figure 3).

### 2.4. Amino Acid Deprivation Abrogates Survival and Cisplatin-Sensitivity of Neuroblastoma Cells

**Figure 4:**
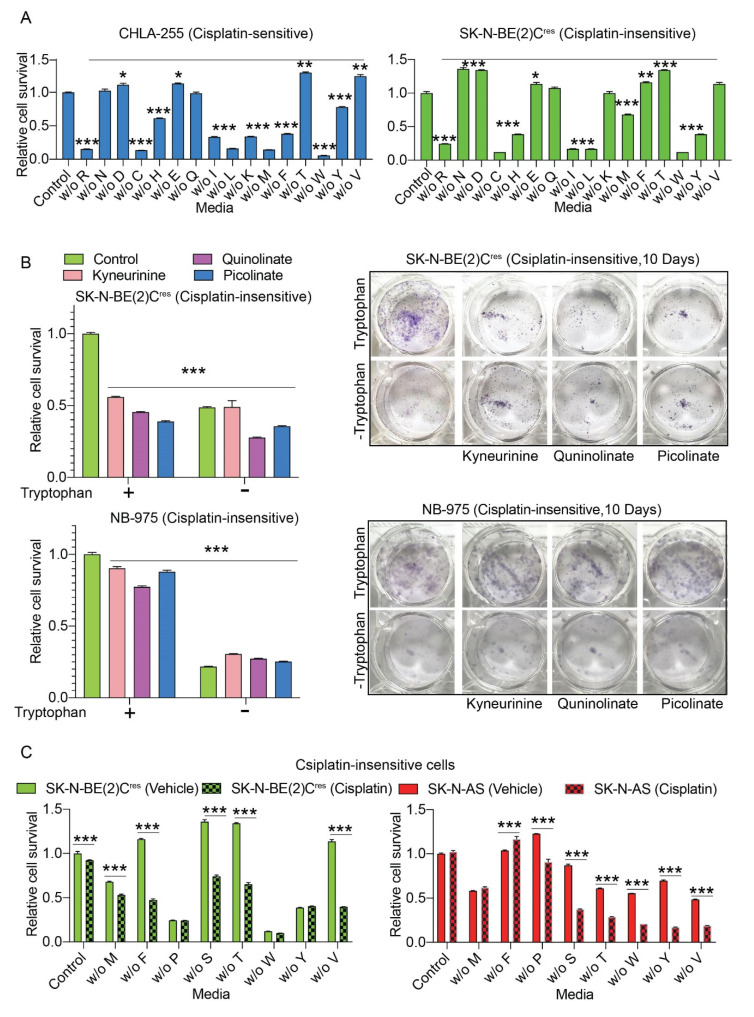
Amino acids regulate cisplatin-sensitivity. (**A**) Bar graphs showing relative change in cell survival of cisplatin-sensitive (CHLA-255) and cisplatin-insensitive (SK-N-AS) and cells cultured in control and amino-acid-deprived media for 72 h. Statistical comparisons were made between control and individual amino acid deprived conditions. Each bar represents mean ± SE of data obtained from three biological replicates. *, ** and *** indicate *p* < 0.05, *p* < 0.01 and *p* < 0.001, respectively, which were obtained through Student’s *t*-test. (**B**) Left panel: Relative change in survival of cells cultured for 72 h in media constituted either with or without Tryptophan and Tryptophan metabolites. Each bar represents mean ± SE of data obtained from three biological replicates. *** indicates *p* < 0.001 obtained through Student’s *t*-test by comparing survival in control media with the survival in modified media as shown in legend. Right panel: Images of cells cultured for 10 days according to conditions maintained in left panel of (**B**). (**C**) Bar graphs showing changes in cell survival of cisplatin-insensitive (SK-N-BE(2)C^res^ and SK-N-AS) cells treated with cisplatin for 72 h in control and amino acid deprived media. Each bar in the graph represents mean ± SE of data obtained from either three biological replicates. *** indicate *p* < 0.001 obtained through One-way ANOVA and Sidak’s multiple comparisons test. Standard single letter amino acid codes were used to represent the amino acid labels in (**A**,**C**).

Upregulation of amino acids in CDDP-insensitive cells prompted us to evaluate the role of amino acids in the survival response of neuroblastoma cells. We prepared cell culture media with or without (w/o) individual essential amino acids, including Arginine (R), Cysteine (C), Isoleucine (I), Leucine (L), Lysine (K), Methionine (M), Phenylalanine (F), Tryptophan (W), Tyrosine (Y), and Tryptophan (W). Cell survival data derived from neuroblastoma cells cultured in either regular or medium-lacking individual amino acids are presented in Figure 4A. Deprivation of most of the essential amino acids resulted in significant reduction of cell survival in CDDP-sensitive (CHLA-255) and CDDP-resistant [SK-N-B(E)2C^res^] cells (Figure 4A). We also evaluated the survival of cisplatin-resistant, human SK-N-BE(2)C^res^ and murine NB-975 cells in Tryptophan-catabolite supplemented media, since Tryptophan derived metabolites were upregulated in CDDP-insensitive cells (Figure 3). Both the cisplatin-insensitive, SK-N-BE(2)C^res^ and murine NB-975 neuroblastoma cells showed reduction in cell survival in 72 h and ten-day cultures performed in Tryptophan-deprived media (Figure 4B). Interestingly, culturing SK-N-BE(2)C^res^ cells in Tryptophan catabolites supplemented media reduced the survival of these neuroblastoma cells in the long-term (10 days) cultures, indicating a negative effect of Tryptophan catabolites on cell survival (Figure 4B). From the experimental data we concluded that the reduction in cell survival observed in neuroblastoma cells as evident in our amino acid deprived conditions was a cumulative effect of a decrease in multiple amino acid derivatives rather than individual catabolites of amino acids.

Further, we evaluated the role of amino acids on CDDP-sensitivity through cell survival analysis using two cisplatin-insensitive cell lines; SK-N-AS that we applied for metabolomic analysis and SK-N-BE(2)C^res^ cells. We observed that deprivation of specific amino acids sensitized CDP-insensitive, SK-N-AS and SK-N-BE(2)C^res^ neuroblastoma cells to CDDP (Figure 4C). Culturing both the CDDP-insensitive cells with a fixed dosage of CDDP concentration less than the IC_50_ for each cell line, in media deprived of individual amino acids, M, S, T, F, P, W, Y, and V reduced the survival of these two cell lines in a significant manner as indicated by the significant differences in survival values with *p* < 0.001(***) obtained through One-way ANOVA and Sidak’s multiple comparisons test shown in Figure 4C.

### 2.5. Hydroxychloroquine Suppresses Amino Acid Metabolism in Cisplatin-Insensitive Cells

**Figure 5:**
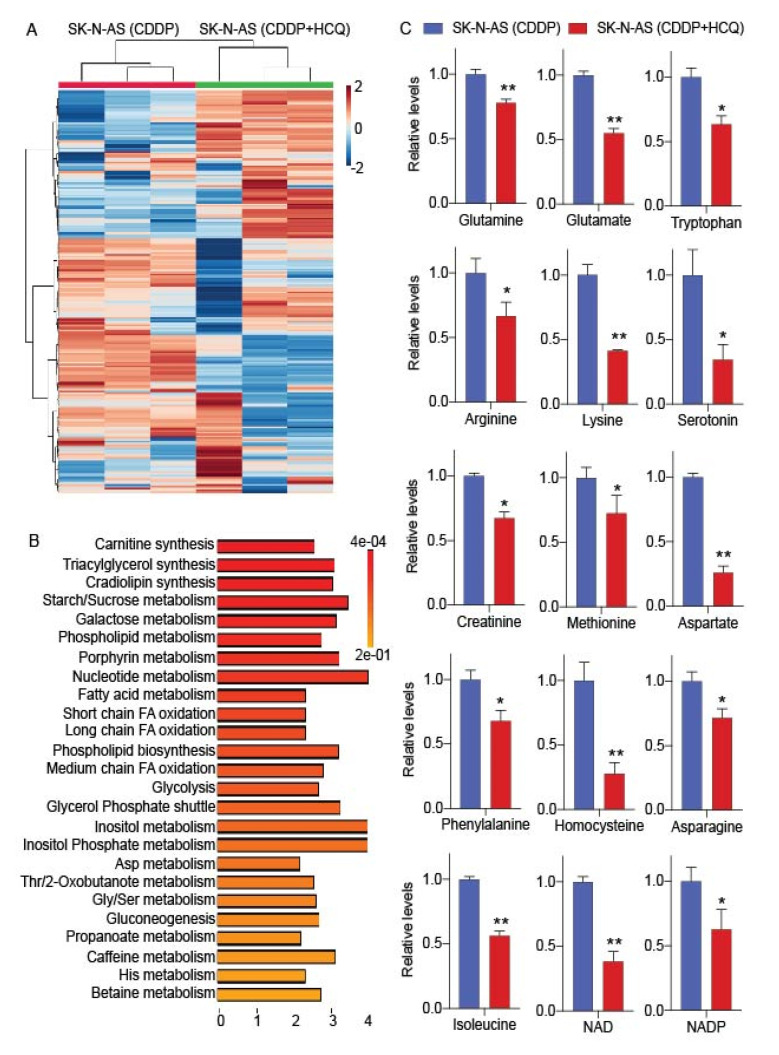
Hydroxychloroquine (HCQ) inhibits cisplatin-induced amino acid metabolism. (**A**) Heatmap showing alterations in metabolic pathways in cisplatin-insensitive cells treated with CDDP and CDDP + HCQ media for 24 h. Colored cells in row represent the alterations in individual metabolites. Scale for heatmap is shown adjacent to the heatmap; (**B**) pathway impact analysis showing significantly impacted pathways in HCQ + CDDP treated cells in comparison to CDDP treated SK-N-AS cells. Scale under the impact plot shows the Fold enrichment and the color scale adjacent to pathway plot indicates the *p*-values for impacted pathways. Standard amino acid codes were used to represent the amino acid labels in this analysis, and (**C**) bar graphs showing relative decrease in amino acids and their metabolites in SK-N-AS cells treated for 24 h in specified conditions. Each bar in the graph represents mean ± SE of data obtained from 3 biological replicates. * and ** represent a *p* < 0.05 and *p* < 0.01, respectively, obtained through Student’s *t*-test. NAD, Nicotinamide adenine dinucleotide; NADP, Nicotinamide adenine dinucleotide phosphate.

Our cell survival analyses indicated that exogenous amino acids were regulating survival, as well as CDDP-sensitivity, in neuroblastoma cells (Figure 4C). In addition to exogenous amino acids, autophagy-mediated recycling of amino acids also facilitates the maintenance of amino acids in cells subjected to stress conditions. Therefore, we applied hydroxychloroquine (HCQ) for inhibiting amino acid metabolism in cisplatin-insensitive neuroblastoma cells based on the premise that HCQ inhibits terminal phase of autophagy, recycling of cellular receptors involved in nutrient uptake, as well as mTOR pathways. For identifying the effect of HCQ on metabolism of drug insensitive cells, we compared the metabolite profiles of SK-N-AS cells treated with CDDP alone and in combination with HCQ. The heatmap derived from metabolite pools of CDDP and CDDP + HCQ treated cells indicated that the metabolite profile of CDDP treated SK-N-AS cells were different from HCQ + CDDP treated cells (Figure 5A). HCQ and CDDP combined treatment also affected the metabolic pathways compared to CDDP treatment alone, in CDDP-insensitive cells as shown in Figure 5B. We analyzed the relative levels of individual amino acids in CDDP alone with HCQ + CDDP treated SK-N-AS cells. Our analyses showed that the relative levels of amino acids and the metabolites derived through amino acid metabolism were reduced significantly in HCQ + CDDP treated conditions compared to CDDP alone treated conditions (Figure 5C). Thus, we concluded that HCQ suppressed amino metabolism in CDDP treated, insensitive, SK-N-AS cells.

### 2.6. Hydroxychloroquine Sensitizes Neuroblastoma Cells to Cisplatin

**Figure 6:**
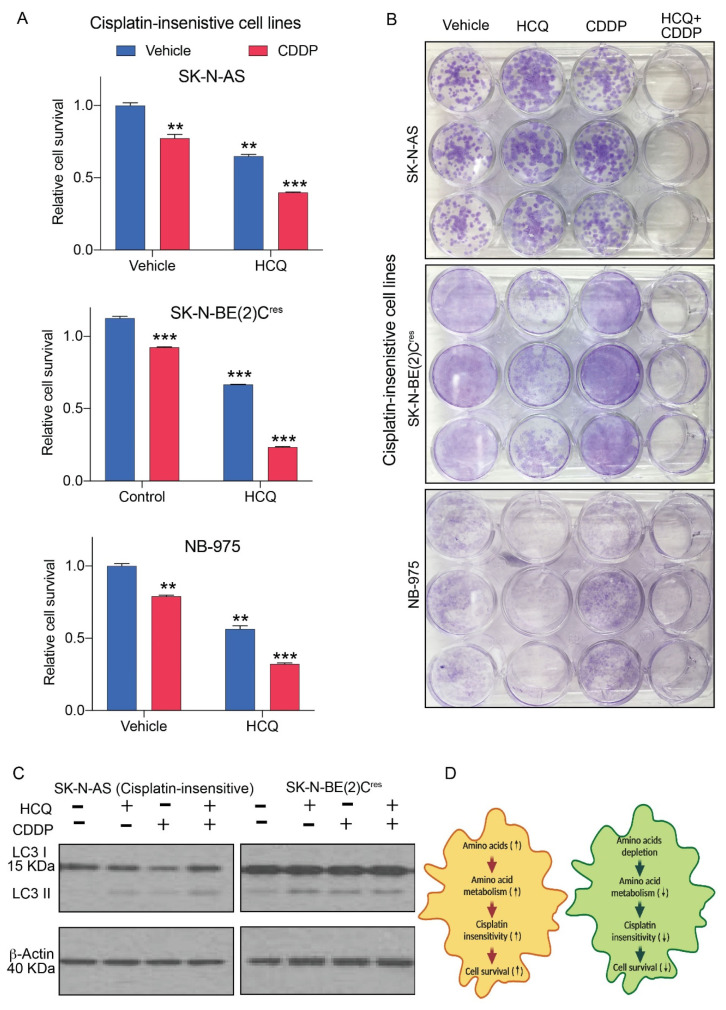
Hydroxychloroquine sensitizes neuroblastoma to cisplatin. (**A**) Bar graphs showing relative change in cell survival of CDDP-insensitive cells in the vehicle, HCQ, CDDP, and combination treatments for 72 h. Each bar in the graph represents mean ± SE of data obtained from 3 or 4 biological replicates. ** and *** represent a *p* < 0.01 and *p* < 0.001, respectively, obtained through Student’s *t*-test. (**B**) Pictures showing changes in survival of CDDP-insensitive cells in the vehicle, HCQ, CDDP, and combination treatments after ten-day treatment with conditions shown in panel. (**C**) Western blots showing changes in LC3I/II band intensities in control and drug-treated CDDP-insensitive cells. β-Actin was used as a loading control. (**D**) Schematic model summarizing that importance of amino acid metabolism in promoting cisplatin-resistance and survival in NBL.

Our metabolomic analyses revealed upregulation of amino acid metabolism in cisplatin-treated, insensitive cells (Figure 3) and suppression of amino acid metabolism in insensitive cells by combination of CDDP and HCQ treatment (Figure 5C). We further evaluated the effects of HCQ on expression patterns of microtubule-associated proteins IA/IB-light chain 3 (*LC3*), in cisplatin-insensitive, neuroblastoma cells upon HCQ and CDDP treatment. We observed that treatment with CDDP reduced LC3 I in CDDP-insensitive SK-N-AS cells. Interestingly, this effect was reversed upon treatment with the autophagy inhibitor, HCQ (2.5 µM), in combination with CDDP (Figure 6A). Another CDDP-resistant neuroblastoma cell line, SK-N-BE(2)C^res^ showed an increase of LC3 II band upon treatment with HCQ, indicating that application of HCQ alters the protein levels of LC3 II upon HCQ treatment in these cells. We further evaluated the survival of neuroblastoma cells in HCQ and CDDP combinations. Cell survival assays performed for 72 h indicated that treatment with HCQ (2.5 µM) reduced cell survival in neuroblastoma cells when treated with HCQ alone or in combination with CDDP (Figure 6B). Treatment of CDDP-insensitive cells with HCQ (1.25 µM) for ten days also reduced the survival of SK-N-BE(2)C^res^ and NB-975 cells. Furthermore, combination treatments, including HCQ and sub-lethal concentrations of CDDP, inhibited the survival of SK-N-AS, SK-N-BE(2)C^res^, and NB-975 cells in long term survival assays (Figure 6C).

## 3. Discussion

Metabolic alterations were identified as one of the hallmarks of cancer development [34]. Recent studies established neuroblastoma as metabolic-driven cancer and enumeration of metabolic deregulated pathways in neuroblastoma are providing novel targets for suppressing the growth of these tumors [35,36,37]. Metabolic pathways including polyamine metabolism [36], single carbon metabolism [30], alanine, aspartate, and glutamate metabolism [38], glycolysis [39] and glutathione metabolism [40], were shown to promote oncogenic growth in neuroblastoma. In addition to supporting tumors’ initiation and growth, tumor-promoting metabolic pathways also play a crucial role in abrogating the therapy response of tumors. Most of the neuroblastoma patients receiving cisplatin in their chemotherapy regimens develop resistance to CDDP. Enumeration of mechanisms leading to CDDP-resistance has shed light on the possible role of diverse oncogene-driven pathways causing CDDP-resistance in neuroblastoma [41]. Despite the well-established and notorious role of metabolic deregulation in neuroblastoma, the contribution of metabolic pathways in CDDP-sensitivity still remains elusive.

Our metabolomic analyses provided an unbiased metabolic phenotype of CDDP-insensitive cells compared to CDDP-sensitive cells. Our study demonstrates that treatment with CDDP alters the metabolism in cisplatin-treated insensitive cells with which exhibit high metabolic plasticity, especially by upregulating their amino acid metabolism in drug treated conditions (Figure 1, Figure 2 and Figure 3). Metabolic plasticity enables cancer growth by promoting tumor cell survival [42]. Especially, amino acids plays a vital role in intermediary metabolism by donating carbon, amino, and amido groups required for metabolic flux, including the nucleotide metabolism in proliferating cells [29,43]. Thus, we envisioned that the upregulation of amino acid metabolism noted in CDDP-treated, insensitive cells could support CDDP-resistant, cellular metabolism in CDDP-insensitive cells (Figure 3). We subsequently confirmed the dependence of neuroblastoma cells on amino acids through the survival decrease observed in CDDP-insensitive cells cultured in media deprived of exogenous amino acids (Figure 4A). Furthermore, amino acid deprivation reduced the survival of CDDP-insensitive cells, similar to the CDDP-sensitive cells in drug-treated conditions (Figure 4C) indicating that high amino acid levels are essential for the survival of cisplatin-insensitive cells in the drug treated conditions. Tryptophan metabolism plays a key role in tumor cell metabolism and stem cell maintenance [44]. In our study, tryptophan catabolites could not support the survival recovery of cisplatin-resistant, human SK-N-BE(2)C^res^ cells, indicating that the amino acid-dependent survival response in neuroblastoma cells is driven cumulatively by multiple amino acid catabolites. Thus, we concluded that mechanisms maintaining amino acid pools were warranted for the survival and CDDP-insensitivity in neuroblastoma cells.

Amino acid metabolism is regulated through the cellular uptake of amino acids, mTOR-mediated signaling, and autophagy-mediated degradation of proteins [32,45,46]. Our survival data (Figure 4) indicates a possible role for amino acid uptake mechanisms in prompting survival, as well as cisplatin-insensitivity, in neuroblastoma cells. However, such possibility could not rule out the role of intracellular amino acid replenishing mechanisms, such as autophagy, in cisplatin-insensitive neuroblastoma cells. Previous studies have established that CDDP treatment induces autophagy in cancer cells [47]. Our strategic application of HCQ, which was shown to inhibit metabolism related pathways, such as autophagy, cellular receptor recycling, and augment mTOR inhibition [48,49], reduced amino acid metabolism of CDDP-insensitive, SN-K-AS cells (Figure 5C). Furthermore, the combination of HCQ and CDDP sensitized CDDP-insensitive SK-N-AS cells in a significant manner (Figure 6A,B) indicating that decrease in relative levels of essential amino acids in HCQ treatment suppressed the survival of cisplatin insensitive SK-N-AS, SK-N-BE(2)C^res^ and NB-975 cells. Amino acids, such as glutamine, required for the nucleotide metabolism, fatty acid metabolism, and the tricarboxylic acid cycle (TCA) cycle [50,51]; tryptophan-essential for protein synthesis, serotonin, and nicotinamide adenine dinucleotide (NAD) generation [52]; arginine and creatinine essential for maintaining the urea cycle [53]; and other essential amino acids were reduced in HCQ + CDDP treated cells, demonstrating suppression of amino metabolism in HCQ drug insensitive, SK-N-AS cells. Furthermore, an increase in LC3 II protein levels in HCQ treated insensitive neuroblastoma cells (SK-N-AS in Figure 6C), along with their decreased survival in HCQ + CDDP treated conditions, indicates that HCQ suppressed amino acid metabolism in CDDP-treated insensitive cells (Figure 5 and Figure 6). Thus, we concluded that metabolic inhibition by HCQ promotes CDDP-sensitivity in neuroblastoma cell lines. However, validation of our findings with other cell lines would provide additional insights on cisplatin resistance mechanisms against neuroblastoma.

Frequent tumor recurrence and poor survival response observed CDDP treated, neuroblastoma patients warrant the need for identifying mechanisms underlying CDDP resistance in neuroblastoma. Omics-based approaches have complemented the identification of mechanisms driving CDDP-resistance in neuroblastoma [54]. Utilizing the metabolomics approach, we provide direct evidence for the possibility of metabolic plasticity in CDDP-insensitive neuroblastoma cells by enumerating the upregulation of amino acids metabolism in cisplatin-insensitive neuroblastoma models. Deprivation of amino acids through dietary regulation and application of enzymes, such as Asparaginase, have been in vogue in cancer therapies [55,56]. Nevertheless, tumor cells regulate their amino acid pools through autophagy mediated recycling which provides alternative route of amino maintenance in tum or cells [57]. Thus, inhibition of both amino acid availability and autophagy mediated process would suppress the amino acid mediated survival advantage in tumor cells. We successfully applied the strategy of sensitizing CDDP-insensitive neuroblastoma cells to sub-lethal doses of CDDP with amino acid deprivation and HCQ treatment. Our study not only confirmed the roles of amino acids in mediating CDDP-insensitivity but also demonstrates that HCQ treatment sensitizes neuroblastoma to CDDP treatment through suppressing amino acid metabolism. Further mechanistic studies elaborating the role of mTOR mediating signaling and autophagic flux in CDDP treated neuroblastoma cells are warranted to establish the role mechanisms regulating amino acid signaling and autophagy in drug resistance models of neuroblastoma.

## 4. Materials and Methods

### 4.1. Neuroblastoma Cell Lines and Culture Conditions

Neuroblastoma cell lines SK-N-BE(2)C, NB-975, SK-N-AS, CHLA-255, SK-N-DZ, and SK-N-BE(2)C^res^ (which is a CDDP-resistant cell line derived from the parental SK-N-BE(2)C parent cell line in our lab) were cultured with media according to American Type Culture Collection (ATCC) guidelines in media containing heat-inactivated fetal bovine serum (FBS, Sigma-Aldrich, St. Louis, MO, USA) and 37 °C in a 5% CO_2_ humidified atmosphere. Media were also supplemented with 50 U of penicillin per mL, 0.1 mg of streptomycin per mL, L-glutamine, sodium pyruvate, and non-essential amino acids as previously described [58].

### 4.2. Cellular Survival Assays

The reagents used for cell survival assays, including CDDP (NDC 0703-5747-11), HCQ sulfate (HCQ, Sigma-Aldrich), Crystal violet (Fisher Scientific, Ward Hill, MA, USA), Tryptophan, Kynurenine, Picolinic acid, Quinolinic acid (Sigma-Aldrich), and 3-(4,5-dimethylthiazol-2-yl)-2,5-diphenyl tetrazolium bromide (MTT, BIOSYNTH International Inc. San Diego, CA, USA), were dissolved and stored according to the manufacturer’s instructions. Cell survival assays for short-term survival were performed in 96-well plates. Briefly, neuroblastoma cells were seeded at a density of 2000 cells per well and cultured overnight before changing to media containing dilutions of either CDDP to required final concentrations or media deprived of individual amino acids and cultured in 37 °C in a 5% CO_2_ humidified atmosphere for 72 h. Post-treatment, MTT was added to media, cells were lysed in dimethyl sulfoxide (DMSO, Fisher Scientific), and cell survival was evaluated through the absorbance values at 570 nm. For the long-term survival assays (ten days), 100–250 cells were seeded in 12-well plates and were cultured in control, drug-containing (unless specified CDDP concentrations below IC_50_ values were applied in long-term cultures), and the media was reconstituted with deprivation of amino acids or supplemented with amino acid metabolites and cultured for ten days. Post-culturing for ten days, media were aspirated from well plates, cells were rinsed and fixed in Methanol (Fisher Scientific), and were further stained with Crystal violet.

### 4.3. Apoptosis Assay

CHLA-255, SK-N-AS, SK-N-BE(2)C and SK-N-BE(2)C^res^ cells were treated in triplicates with either vehicle or 500 nM Csiplatin for 24 h. We trypsinized the treated cells and analyzed for the apoptotic and necrotic populations using the APC AnnexinV apoptosis detection kit with 7-AAD (#640930, BioLegend, San Diego, CA, USA), according to the manufacturer’s instructions. Flow cytometry analyses were performed using the BD SLR II flow cytometer (BD Biosciences, Franklin Lakes, NJ, USA).

### 4.4. Polar Metabolome Analysis

CHLA-255 and SK-N-AS cells were cultured in different conditions containing vehicle, CDDP (sub-lethal doses), or HCQ + CDDP containing DMEM (Corning, Corning, NY, USA) with 10% FBS for 24 h. Cells were rinsed and extracted with 80% ice-cold methanol on dry ice for polar metabolites. Targeted metabolomic data analysis was performed as described previously [59].

### 4.5. Western Blot Analysis and Antibodies

Rabbit Polyclonal anti-LC 3 (14600-1-AP) and rabbit polyclonal anti-beta Actin (20536-1-AP) were purchased from Proteintech (Rosemont, IL, USA). The cell lysate preparation and immunoblotting were performed using methods described before [10].

### 4.6. Statistical Analysis

The data in metabolite and cell survival analyses were expressed as mean ± standard error (SE). The difference between the control, amino acid-deprived, and treatment groups were analyzed using Student’s t-test, considering the control and treated groups as two groups. The data analysis was carried out using Microsoft Excel and GraphPad Prism 7.0 software (Prism-GraphPad, San Diego, CA, USA).

## 5. Conclusions

Our results delineate the role of amino acid metabolism in cisplatin-insensitive models of neuroblastoma. We provide a direct metabolite read out of amino acid upregulation in CDDP-insensitive cells which get suppressed upon treatment with hydroxychloroquine. While the molecular mechanisms leading to upregulation of amino acid pools in CDDP-insensitive models are yet to be identified, our study provides novel insights into the role of amino acid metabolism in regulating CDDP response of neuroblastoma.
